# *TC003132* is essential for the follicle stem cell lineage in telotrophic *Tribolium* oogenesis

**DOI:** 10.1186/s12983-017-0212-2

**Published:** 2017-05-19

**Authors:** Matthias Teuscher, Nadi Ströhlein, Markus Birkenbach, Dorothea Schultheis, Michael Schoppmeier

**Affiliations:** 1 Department Biology, Developmental Biology Unit, Friedrich-Alexander-University Erlangen-Nuremberg, Staudtstr. 5, 91058 Erlangen, Germany; 2 Present address: Institute of Neuropathology, University Hospital Erlangen, Schwabachanlage 6, 91054 Erlangen, Germany

**Keywords:** *Tribolium*, telotrophic oogenesis, follicle stem cells, Notch-signalling, large-scale RNAi screen, iBeetle

## Abstract

**Background:**

Stem cells are undifferentiated cells with a potential for self-renewal, which are essential to support normal development and homeostasis. To gain insight into the molecular mechanisms underlying adult stem cell biology and organ evolution, we use the telotrophic ovary of the beetle *Tribolium.* To this end, we participated in a large-scale RNAi screen in the red flour beetle *Tribolium*, which identified functions in embryonic and postembryonic development for more than half of the *Tribolium* genes.

**Results:**

We identified *TC003132* as candidate gene for the follicle stem cell linage in telotrophic *Tribolium* oogenesis. *TC003132* belongs to the Casein Kinase 2 substrate family (CK2S), which in humans is associated with the proliferative activity of different cancers. Upon *TC003132* RNAi, central pre-follicular cells are lost, which results in termination of oogenesis. Given that also Notch-signalling is required to promote the mitotic activity of central pre-follicular cells, we performed epistasis experiments with *Notch* and *cut*. In addition, we identified a putative follicle stem cell population by monitoring the mitotic pattern of wild type and *TC003132* depleted follicle cells by EdU incorporations. In *TC003132* RNAi these putative FSCs cease the expression of differentiation makers and are eventually lost.

**Conclusions:**

*TC003132* depleted pre-follicular cells neither react to mitosis or endocycle stimulating signals, suggesting that *TC003132* provides competence for differentiation cues. This may resemble the situation in *C. elegans* were CK2 is required to maintain the balance between proliferation and differentiation in the germ line. Since the earliest effect of *TC003132* RNAi is characterized by the loss of putative FSCs, we posit that *TC003132* crucially contributes to the proliferation or maintenance of follicle stem cells in the telotrophic *Tribolium* ovary.

**Electronic supplementary material:**

The online version of this article (doi:10.1186/s12983-017-0212-2) contains supplementary material, which is available to authorized users.

## Background

In *Drosophila*, germline and follicle stem cells work in a coordinated fashion to produce egg chambers [1, 2]. Two to three germline stem cells (GSC) reside at the anterior tip of the germarium and divide asymmetrically to give rise to a cystoblast, which undergoes four mitotic divisions with incomplete cytokinesis to form an interconnected 16-cell cluster. One of these 16 cells is specified as an oocyte and the other cells differentiate into polyploid nurse cells that support the growth of the oocyte. These clusters are engulfed in the germarium by stromal escort cells (EC) until they reach the region 2a/2b border where the two somatic follicle stem cells (FSC) are located, which are maintained in part by niche signals from escort cells [3–5]. Initially, FSC daughter cells are unspecified and have the potential to either cross-migrate to the opposite side and replace the other stem cell or differentiate into either main body or polar/stalk precursors [6–8]. Notch, as well as JAK-STAT signalling further subdivides the stalk/polar pre-follicular cell linage in a stepwise manner [9–11]. Polar cell fate is induced in a restricted subset of this population by the Notch ligand Delta, which is produced in germline cells and induces the adjacent anterior follicle cells to differentiate into polar cells. These cells subsequently express the JAK/STAT ligand Unpaired, inducing stalk cell fates [9, 12]. Therefore three distinct follicle cell populations are present after follicle budding: polar cells which act as signalling centres, stalk cells that connect adjacent egg chambers, and main-body follicle cells which form an epithelium overlying the germline cyst [13].

In contrast to polytrophic-meroistic oogenesis represented by *Drosophila*, in telotrophic-meroistic *Tribolium* oogenesis oocytes and nurse cells of a germ cell cluster separate in a way, in which each follicle contains only one germ cell, the oocyte. Oocytes remain connected to the tropharium – a syncytium of nurse cells – by a nutritive cord [14]. In *Tribolium,* germline proliferation is restricted to larval and early pupal stages, whereas the FSC niche remains active up to adulthood [14]. Thus, the formation and maintenance of the follicle stem cell (FSC) linage in *Tribolium* is largely independent of the germline stem cells (GSCs).

In the *Tribolium* ovary, arrested pro-oocytes are arranged around the somatic plug, a group of small somatic cells located at the posterior end of the tropharium. Upon maturation, pro-oocytes separate from the somatic plug and enter the vitellarium, where they come in contact with pre-follicular cells, which successively encapsulate the oocyte to form an egg-chamber [15, 16]. Previously, we showed that Notch-signalling is required for encapsulation and early steps in follicle cell patterning, i.e. the determination of terminal follicle cells [15]. Subsequently, graded levels of JAK-STAT signalling specify additional follicle sub-populations, including stalk precursor cells. Upon JAK-STAT RNAi, stalk cells are absent and anterior and posterior follicle cells of adjacent vitellogenic egg chambers maximise their area of contact, resulting in severe deformation of follicles [16]. During pre-vitellogenic growth, *Tribolium* oocytes increase in size, while follicle cells divide to form a uniform epithelium surrounding the oocyte [16]. Subsequently, follicle cells enter endocycle and eventually secrete the eggshells. However, in contrast to *Drosophila* where a Notch signal induces the follicle cells to leave mitosis [17, 18], in *Tribolium* egg-chambers Notch signalling prevents follicle cells from entering endocycle prematurely. Hence, with respect to the cycle/endocycle switch, Notch-signalling in *Tribolium* and *Drosophila* has opposing effects [15]. While polytrophic and telotrophic oogenesis may involve the stepwise specification of follicle cell populations in a JAK-STAT and Notch dependent manner [15, 16], the regulatory mechanisms that determine and maintain the follicle stem cell lineage in telotrophic *Tribolium* oogenesis remains to elucidated.

In order to gain additional insights into the molecular mechanisms underlying telotrophic oogenesis and somatic stem cell biology, we participated in the iBeetle screen. The iBeetle screen was a large-scale RNAi screen in *Tribolium*, which identified functions in embryonic and postembryonic development for more than half of the *Tribolium* genes [19]. Here we report on the identification of the putative CK2 substrate *TC003132*. We show that *TC003132* crucially contributes to the specification of the follicle stem cell linage in the telotrophic *Tribolium* ovary.

## Methods

### Strains

The initial phenotype for *TC003132* (iB_00521) was found and reproduced in the Pig-19 [20] strain of *Tribolium castaneum*. All subsequent experiments were performed in the wild type strain San Bernadino (SB) [21]. No differences in the phenotype due to strain-specific effects could be observed [22]. All beetles were reared under standard conditions [23] on white wheat flour containing 5% dry yeast at 25 °C and shifted to 32 °C for the experiments.

### Dissection and antibody staining

Dissection and fixation of adult ovaries was essentially performed as described previously [14–16] (see also Additional file 1: Methods). To visualize morphology, Hoechst 33,258 (5 μg/ml) and Phalloidin (Atto488 or Atto647N fluorophore) (1:50; Sigma) were used. The anti-β-catenin antibody (gift of Gregor Bucher) was used at a 1:500 dilution. To detect mitotically active cells, an anti-phospho-Histone H3 antibody (rabbit polyclonal, 1:100, Upstate) was used. The crossreacting mouse monoclonal anti-*Drosophila-*Eyes-absent and anti-*Drosophila*-Cut antibodies (eya10H6 and 2B10 respectively, DSHB) were used in a 1:100 dilution. The following secondary antibodies were all used at a 1:200 dilution: Alexa555-conjugated goat anti-mouse (Invitrogen), DyLight649 conjugated goat anti-rabbit (Jackson Immuno-Research).

### EdU incorporation assay

For the EdU (5-ethynyl-2′-deoxyuridine) experiments we used the EdU-Click 488 kit (baseclick GmbH). EdU stock solution was dissolved in water (25 mM). For injections 5 mM dilutions were used. Detection was carried according to the manufacturers protocol. EdU positive cells were counted using the Cell Counter plugin of Fiji [24].

## Results and discussion

### Early follicle cell lineages in *Tribolium*

In *Drosophila*, follicle stem cells (FSC) undergo asymmetric division, and daughter cells are either specified as main-body or as polar/stalk precursors, which is distinguishable by relative expression levels of Castor (Cas) and Eyes absent (Eya) [8]. Both, *Drosophila* Cas and Eya are expressed in FSCs, and as their siblings differentiate, cells either express more Cas and lose Eya, or vice versa. While cells with higher Cas apparently differentiate into polar or stalk cells, the cells in which Eya expression remains at high levels differentiate into main-body follicle cells.

**Fig. 1:**
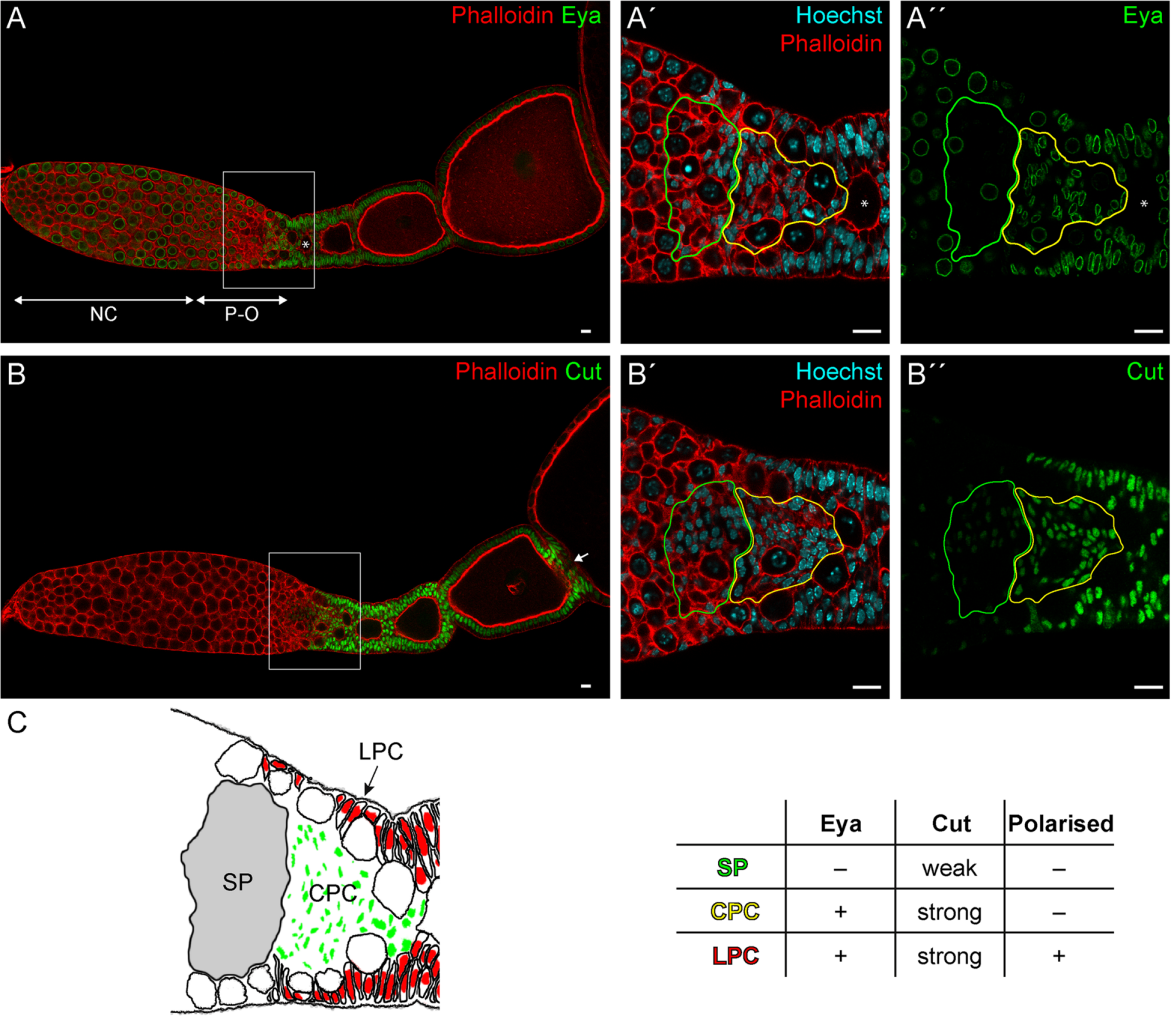
(**A**–**B″**) Detection of Eya (**A**–**A″**) and Cut (**B**–**B″**) (*green*) Phalloidin for f-Actin (*red*) and Hoechst 33,258 for DNA (*blue*) in wild type ovarioles. **A′**–**A″** and **B′**–**B″** are magnifications of *boxed* regions in **A** and **B**, respectively. The *asterisk* in **A**–**A″** marks the first aligned oocyte. (**C**) Schematic representation and features of somatic cell types of boxed regions in **A** and **B**. See text for details. NC: nurse cells; P-O: pro-oocytes; SP: somatic plug; CPC: central prefollicular cells; LPC: lateral prefollicular cells; *green* and *yellow outlines* mark SP and CPCs, respectively. Scale Bar: 10 μm

Previously, we showed that during telotrophic *Tribolium* oogenesis the initial distinction of terminal/stalk precursor cells versus epithelial follicle takes place not until encapsulation [16], raising the questions, to which degree even earlier follicle cell populations can be identified. To this end, we analysed pre-follicular cells in the anterior vitellarium by morphology and cross-reacting antibodies against Eya and Cut (Fig. 1). As shown before [15], Eya is strongly expressed in all mitotically active somatic follicle cells (Fig. 1, A–A″, C) and expression of Eya ceases as follicle cells enter the endocycle. In addition, Eya expression can be observed in germ-line derived cells, including nurse-cells and pro-oocytes (Fig. 1, A). This expression, however, is lost upon entry of pro-oocytes into the vitellarium.

In contrast to Eya, Cut, which in *Drosophila* is required for cell differentiation and tissue homeostasis [25–30], is not restricted to mitotically active cell, as we monitored low levels of Cut expression also in the somatic plug (SP) (Fig. 1, B–B″). The somatic plug consists of small post-mitotic cells and separates the tropharium from the vitellarium (Fig. 1, C) [15]. All pre-follicular and mitotic active follicle cells show strong Cut expression. Again, Cut fades as the cells enter the endocycle. Only terminal follicle cells that separate individual oocytes retain high Cut levels (Fig. 1, B). While somatic plug cells can be distinguished from somatic follicle cells by marker gene expression (i.e. weak Cut expression and absence of Eya), early pre-follicular cell populations reveal distinct differences in morphology and polarisation status (Fig. 1, C). Lateral pre-follicular cells (LPC) are noticeably polarized, while central pre-follicular cells (CPC) do not show a distinct apical-basal polarity. Still, both pre-follicular cell populations show high level of Cut and Eya expression. Thus, irrespective of the less complex egg chamber architecture in telotrophic ovaries, we were able to identify two additional follicle cells subpopulations in *Tribolium*.

### A putative follicle stem cell population

**Fig. 2:**
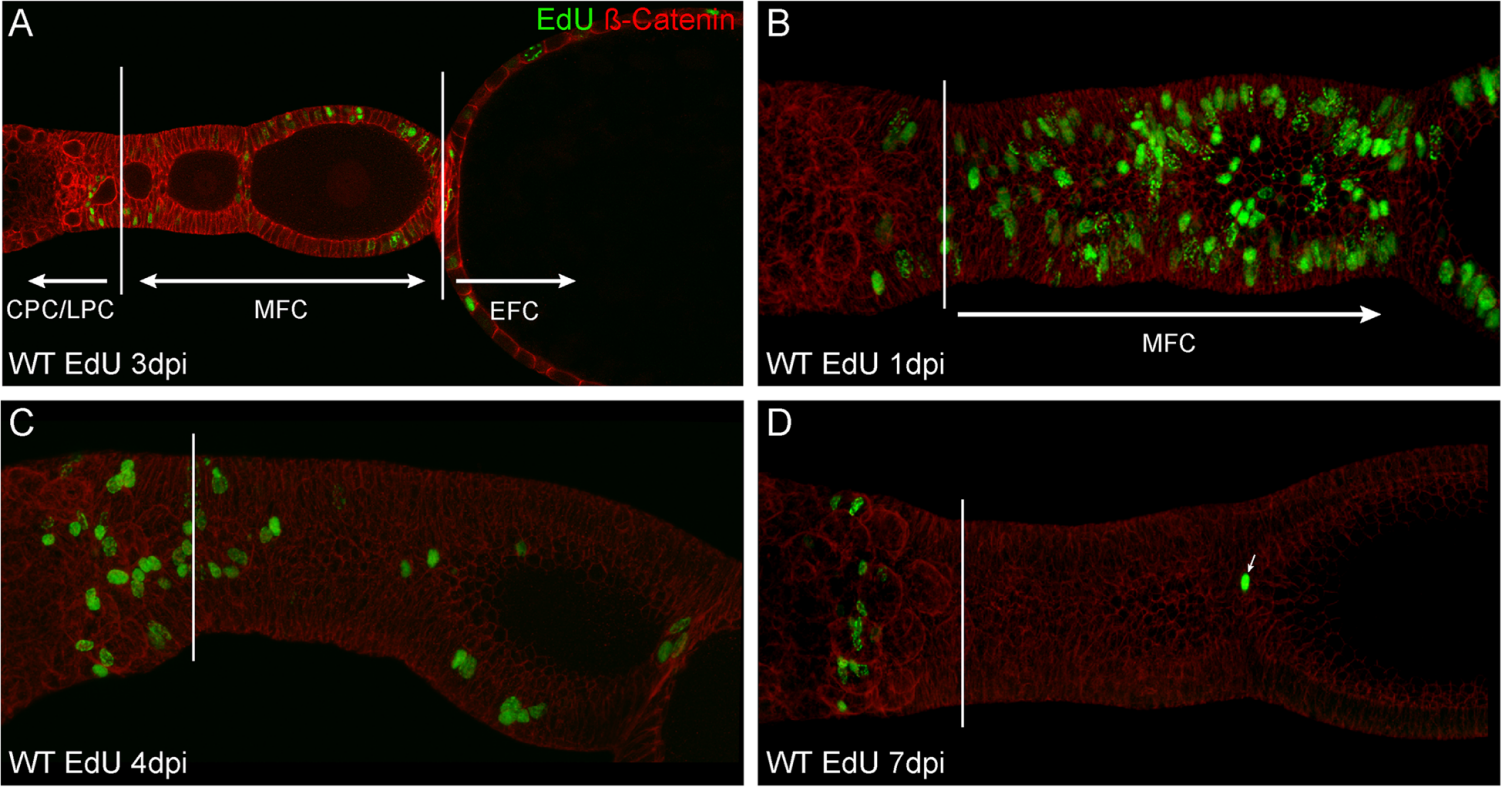
**a**–**d** Detection of EdU (*green*) and ß-Catenin (*red*) in wild type ovarioles. In (**a**) a single focal plane is shown two visualize the distinction between the different cell populations. **b**–**d** are standard deviation projections reflecting total EdU incorporation. The borders between CPCs/LPCs and MFCs are indicated by a *solid white bar*. See text for further details. CPC: central prefollicular cells; LPC: lateral prefollicular cells; MFC: mitotic follicle cells; EFC: endocycling follicle cells; dpi: days post injection

Given that pre-follicular cells in the anterior vitellarium can be further subdivided into central and lateral pre-follicular cells (Fig. 1, C), we asked whether these cells also show pronounced differences in their mitotic pattern. To this end, we adopted pulse-chase incorporation of EdU into ovaries of adult *Tribolium*. EdU (5-ethynyl-2′-deoxyuridine) is an alternative for BrdU (5-bromo-2′-deoxyuridine) assay to directly measure active DNA synthesis or S-phase synthesis of the cell cycle [31, 32]. Five millimeters EDU was injected into adult beetles, ovaries were dissected at 1, 2, 3, 4 and 7 dpi and EdU positive follicle cells were counted (Table 1, Fig. 2, Additional file 1: Table S1). Central and lateral pre-follicular cells were distinguished from mitotic follicle cells after egg-chamber formation (termed “mitotic follicle cells”; MFC) and follicle cells in endocycle (EFC) by morphology and position (Table 1 and Fig. 2a). Already 1 day after injection we found EdU to be effectively incorporated (Fig. 2b; Table 1). At 1dpi more than 592 cells were labelled in average. This number declines to almost zero within a week, as labelled follicle cells proceed with oogenesis and thus, are “used up”. For instance, 314 EdU positive MFC were found 1 day after injection (Table 1, Fig. 2, Additional file 1: Table S1). This declines to 178 at 3dpi. At 4 to 7dpi the number of EdU positive MFC drops to almost zero. Follicle cells in endocycle reach a maximum at 2 days after injection. This population consists of two indistinguishable subpopulations. On the one hand, cells are marked that incorporated EdU into the DNA solely during polyploidisation. On the other hand, cells which have incorporated EdU at earlier time points, i.e. during the S-Phase of Mitosis, should still be detectable, once they started endoreplication. This explains, why observed peak incorporation of EdU in EFCs is delayed compared to the MFCs. Therefore, judging by the incorporation rates of EdU in MFCs, we conclude, that EdU is “used up” 1 to 2 days after injection.

**Table 1:** EdU positive cells in WT

	1dpi	2dpi	3dpi	4dpi	7dpi
CPC	1 ± 1	5.75 ± 2.38	7 ± 3	6.75 ± 2.38	4.75 ± 1.92
LPC	8.25 ± 0.83	13.25 ± 3.83	12 ± 4.85	4.75 ± 1.92	1.5 ± 0.87
MFC	314.75 ± 31.40	305.75 ± 90.34	178.5 ± 87.97	18.25 ± 9.81	0.5 ± 0.5
EFC	268.5 ± 37	308.75 ± 97.87	226.25 ± 59.63	9.25 ± 6.22	0 ± 0

EdU positive cells in follicle cell populations at indicated time-points. Values are given as mean ± standard deviation. *n* = 4; *CPC* central prefollicular cells, *LPC* lateral prefollicular cells, *MFC* mitotic follicle cells, *EFC* endocycling follicle cells, *dpi* days post injection

Strikingly, pre-follicular subpopulations gain or lose the EdU signal at different rates. LPC cells start incorporating EdU already at 1dpi and reach a maximum number at 2dpi. At 7dpi, the number of EdU positive lateral pre-follicular cells drops to almost zero, resembling the overall trend for EdU incorporation. Central pre-follicular cells, however, start incorporating EdU not before 2dpi and moreover, EdU signals persist in a subset of central pre-follicular cells even beyond 7dpi. While the CPCs only account for 0.17% of all marked cells at 1 dpi, they represent over 70% at 7 dpi (Table 1, Fig. 2, Additional file 1: Table S1). Given that the EdU is used up already after 1–2 dpi, our results indicate that these label retaining CPCs represent a previously unknown subpopulation of central pre-follicular cells, dividing at slow rates.

Given that a slow division rate is one the hallmarks of stem cells and that retention of a thymidine analogue has been proven to be indicative for stem cells in various systems [33–37], it is tempting to speculate that these slow dividing cell may represent the follicle stem cell population of the telotrophic ovary.

### *TC003132* is required for early follicle cell specification

To identify genes that are required for the early somatic follicle cell lineage in telotrophic oogenesis, we screened the iBeetle database [38]. The iBeetle screen was a large-scale RNAi screen *Tribolium*, which identified functions in embryonic and postembryonic development for more than half of the *Tribolium* genes [19]. Among others, we identified *TC003132* as candidate gene for early follicle cell patterning. *TC003132* encodes a previously uncharacterized protein, which is widely conserved within insects, but is not found in *Drosophila* [39]. Searching the NCBI Conserved Domain Database [40] revealed that TC003132 belongs to the Casein Kinase 2 substrate (CK2S) family (pfam15011).

**Fig. 3:**
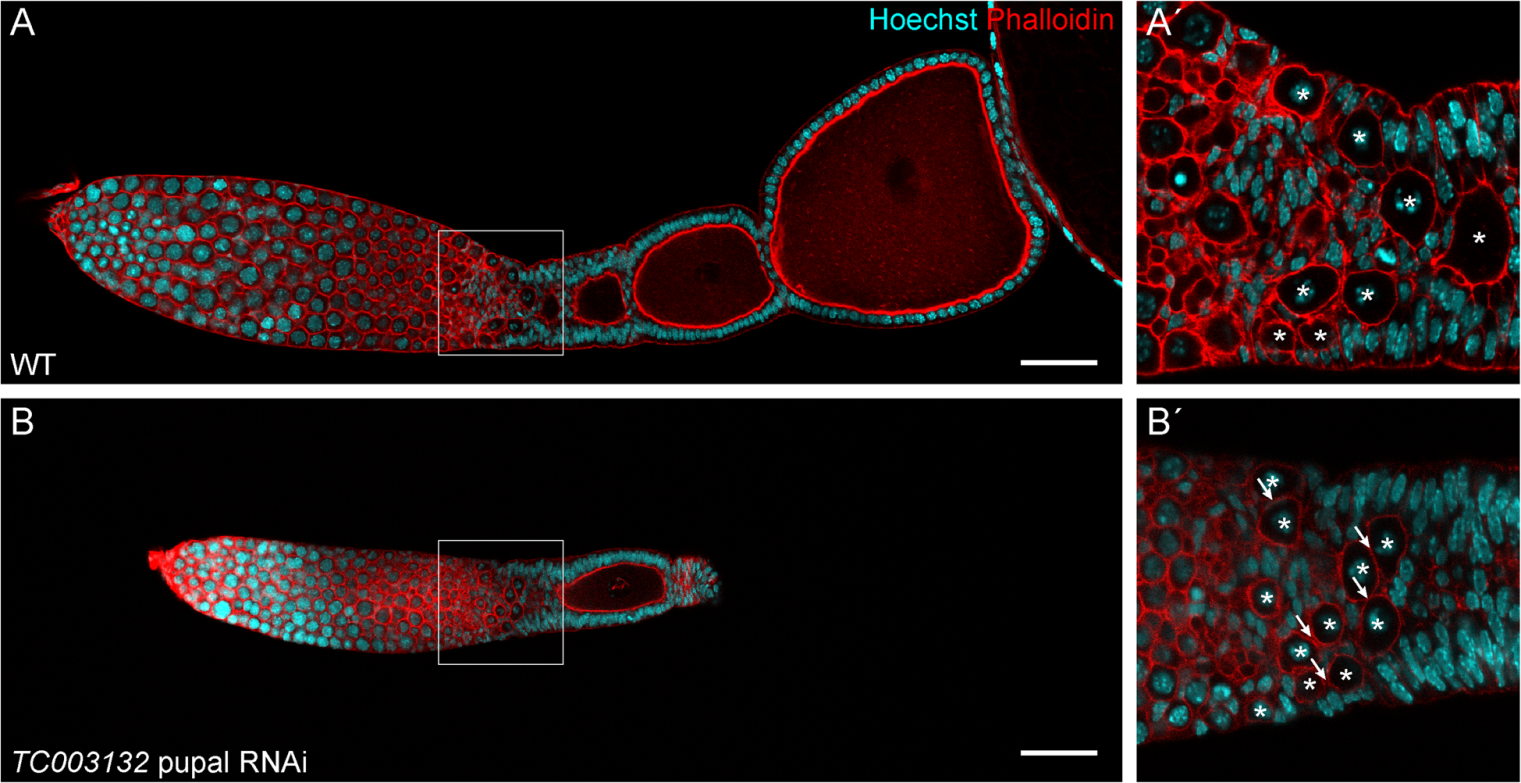
(**A**–**A′**) Wild type ovariole of *Tribolium* stained with Phalloidin for f-Actin (*red*) and Hoechst (*blue*). In wild type, pro-oocytes leave the tropharium, become encapsulated by follicle cells and are aligned in the centre (**A′**, magnification of indicated region in **A**). (**B**–**B′**) Pupal knockdown of *TC003132* at 7dpi. The vitellarium is shortened and only a single growing follicle is obvious (**B**). Oocytes are not longer encapsulated, resulting in oocytes arranged side-by-side (**B′**). *Asterisks* mark oocytes and Arrows point to oocytes contacting each other, thus indicating encapsulation defects. Scale bar in **A** and **B**: 50 μm

Pupal RNAi of *TC003132* results in very severe phenotypes (Fig. 3). As compared to wild type, RNAi ovarioles are depleted of most egg-chambers and the vitellarium is shortened. In addition, the transition zone between tropharium and vitellarium, which harbours the somatic plug, pro-oocytes, and pre-follicular cells, is severely affected (Fig. 3, B–B′). In wild type, arrested pro-oocytes are arranged around the somatic plug. Upon maturation, pro-oocytes separate from the somatic plug and enter the vitellarium, where they come in contact with pre-follicular cells, which successively encapsulate the oocyte to form an egg-chamber [15, 16]. Upon *TC003132* RNAi pro-oocyte maturation and encapsulation is impaired. Pro-oocytes are not longer encapsulated by central pre-follicular cells, but remain in the transition zone, resulting in an excess of pro-oocytes in this region (Fig. 3, asterisks and arrows in B′). Interestingly, while lateral pre-follicular cells are basically present, central pre-follicular cell populations are largely absent (see below).

**Fig. 4:**
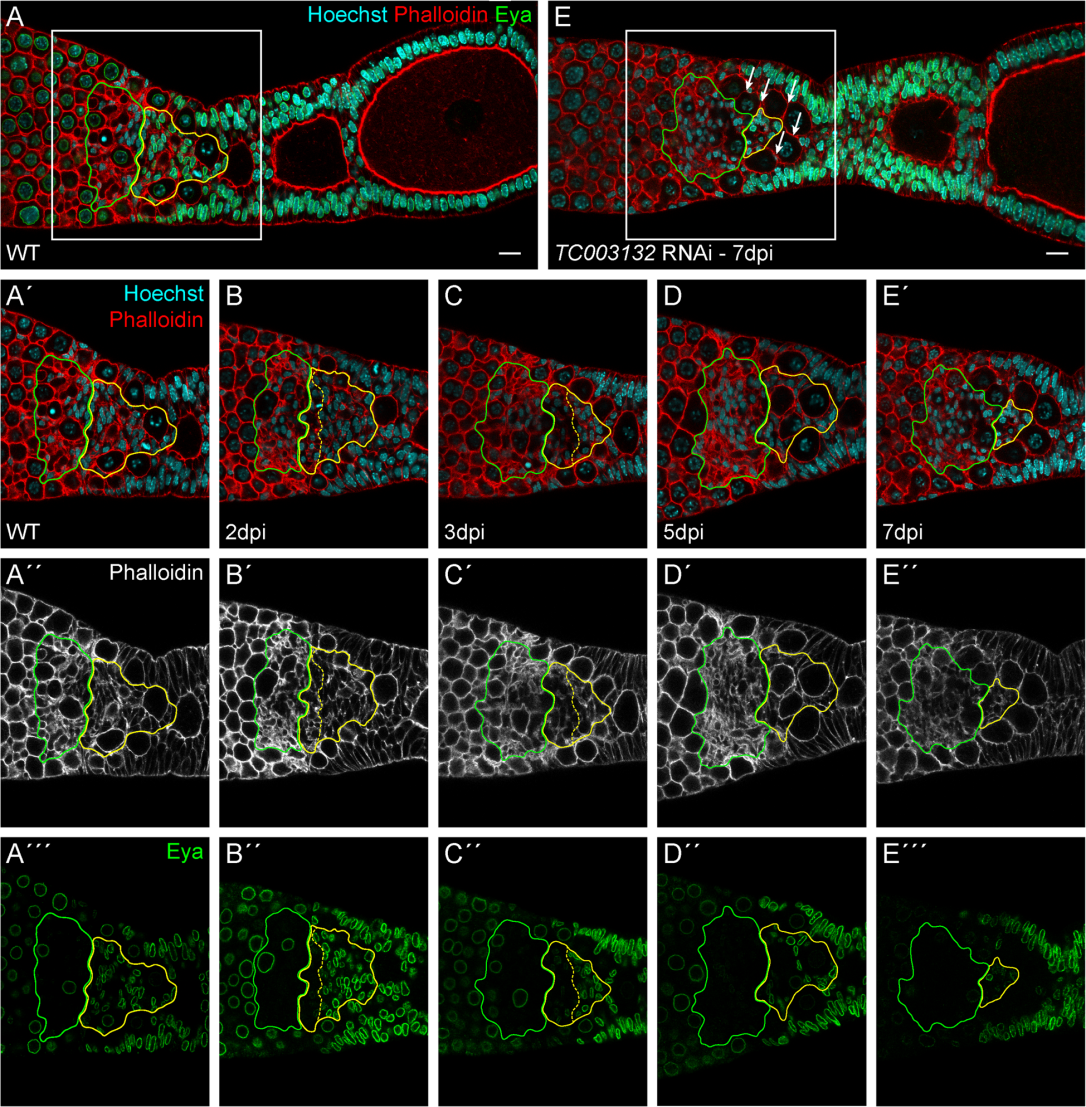
(**A**–**A‴**) Wild type ovariole stained with Phalloidin for f-Actin (red in **A**–**A′**, *white* in **A″**), Eya (*green*), and Hoechst (*blue*). Eya is expressed in central pre-follicular cells (CPC, *yellow outline*) and the somatic plug (SP, *green outline*) (**B**–**E‴**) Ovarioles dissected at indicated time points after adult knockdown of *TC003132*. A subset of CPC starts to express Eya at lower levels (border between high and low expression of Eya in CPCs is marked by the *yellow dashed line*) (**B**–**B″**). At 3dpi, CPCs occupy a smaller area and Eya expression is further reduced. The somatic plug (SP) appears less condensed and occupies a larger area (**C**–**C″**). CPCs expressing reduced amounts of Eya are lost at 5dpi (**D**–**D″**). The terminal phenotype can be observed at 7dpi. Only a small population of Eya positive CPCs is still present and the SP has expanded even more. Also encapsulation defects become apparent as young oocytes can be found in direct contact to each other (*arrows*, **E**–**E‴**). Scale bar: 10 μm

To further elucidate the functions of *TC003132* in early follicle cell specification, we performed adult RNAi and analysed the resulting phenotypes by morphology and Eya staining (Fig. 4). In contrast to pupal RNAi, which affects initial phases of oogenesis, e.g. primary oocyte maturation, encapsulation, or follicle cell patterning, adult RNAi can be utilised to elucidate on-going processes in follicle cell specification and oogenesis [16]. Adult beetles were injected with dsRNA corresponding to *TC003132* and ovaries were dissected at different time points (Fig. 4). Interestingly, we observed effects of *TC003132* depletion even before morphological phenotypes become obvious. Two days after injection, anterior central pre-follicular cells cease Eya expression (Fig. 4, B–B″). At 3dpi, this region of “Eya negative CPC” expands toward the vitellarium and these cells become indistinguishable from the somatic plug (Fig. 4, C–C″). As deduced from morphology, “Eya negative CPC” initially remain in this region and also pro-oocyte maturation and encapsulation is unaffected, Subsequently, however, central pre-follicular cell are lost, encapsulation fails, and pro-oocytes start to accumulate in the transition zone (Fig. 4, C–E). At that time, we also observed effects on the somatic plug (Fig. 4, C′, D′, E″). As compared to wild type, 5 days after *TC003132* RNAi the plug region is less defined and seems to occupy a larger area. Moreover, actin-structures appear to be less condensed (Fig. 4, D′, E″). Seven days after injection, adult RNAi phenotypes basically resemble pupal phenotypes (Fig. 4E–E‴).

**Fig. 5:**
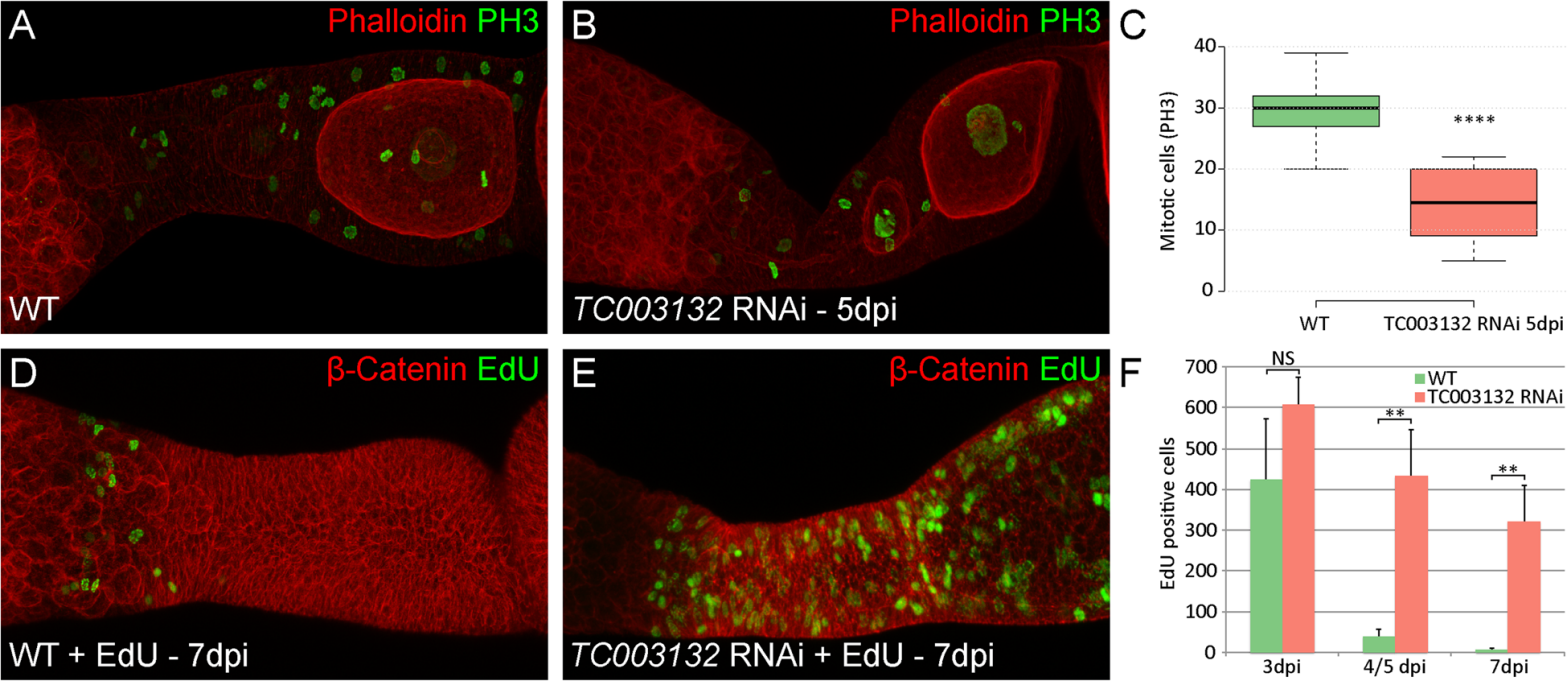
**a**–**b** Staining of phosphorylated histon H3 (PH3, *green*) and Phalloidin (*red*) in wild type and *TC003132* RNAi ovarioles. **c** Box plot of PH3 positive cells in WT (*green*) and *TC003132* RNAi at 5dpi (*red*). Centre lines show the medians; box limits indicate the 25th and 75th percentiles as determined by R software; whiskers extend 1.5 times the interquartile range from the 25th and 75th percentiles. *n* = 13, 14 sample points. **d**–**f** Detection of EdU (*green*) and ß-Catenin (*red*) in wild type and *TC003132* RNAi ovarioles. Compared to wild type, the amount of follicle cells in mitosis is approximately halved 5dpi after *TC003132* RNAi (**a**–**c**). Both, wild type and *TC003132* knockdown exhibit similar amounts of EdU incorporation at 3dpi (**f**). In wild type, the signal is rapidly lost in (**d**, **f**) and only the anteriormost central pre-follicular cells remain EdU positive (**d**). In *TC003132* RNAi, the EdU signal is stable and diminishes at a much slower rate (**e**, **f**). *Error bars* in (**f**) represent standard deviation. Dpi: days post injection; NS: Not significant; ** and **** *P* < 0.01 and *P* < 0.0001 using a two-tailed t-test, respectively

In order to elucidate if the loss of pre-follicular cells in *TC003132* RNAi might be due to a reduction in mitotic activity, we monitored cell division patterns by a cross-reacting anti- body against phosphorylated histone-3 (pH 3) (Fig. 5, Additional file 1: Table S2). In wildtype, an average number of 30 mitotically active cells per ovariole were found (Fig. 5a, c). However, as pH 3 positive cell are distributed rather randomly around the transition zone and vitellarium, there are no distinct domains or patterns of mitotic activity. Five days after *TC003132* depletion, most follicle cells cease mitosis and the number of cell divisions drops to an average number of 14 mitotically active cells per ovariole (Fig. 5b, c). Again, we did not observe any obvious pattern, indicating that *TC003132* RNAi results in the overall reduction of mitosis. This is further supported by EdU incorporation assays. In *TC003132* RNAi ovarioles, EdU signals decrease at significantly slower rates (Fig. 5e–f; Additional file 1: Table S3), confirming a reduced mitotic activity of follicle cells.

Hence, our results suggest that *TC003132* has (at least) a dual function. In the transition zone, it is mainly required for the specification and/or maintenance of central pre-follicular cells. In addition, *TC003132* has a rather global function in promoting mitosis of follicle cells in the vitellarium.

### *Tc-cut* RNAi results in the overproliferation of somatic follicle cell lineages

Given that that *TC003132* is required to promote mitotic activity of the somatic follicle stem cell linage in *Tribolium*, we asked whether *TC003132* function depends on Notch-signalling and/or *cut* action. Previously, we showed that Notch-signalling in *Tribolium* is among others required to maintain the mitotic cycle of somatic follicle cells [15]. In *Drosophila*, the mitosis/endocycle switch is mediated by Notch-depended down-regulation of *cut* expression and the loss of *Drosophila cut* function results in premature entry of main-body follicle cells into the endocycle [30].

**Fig. 6:**
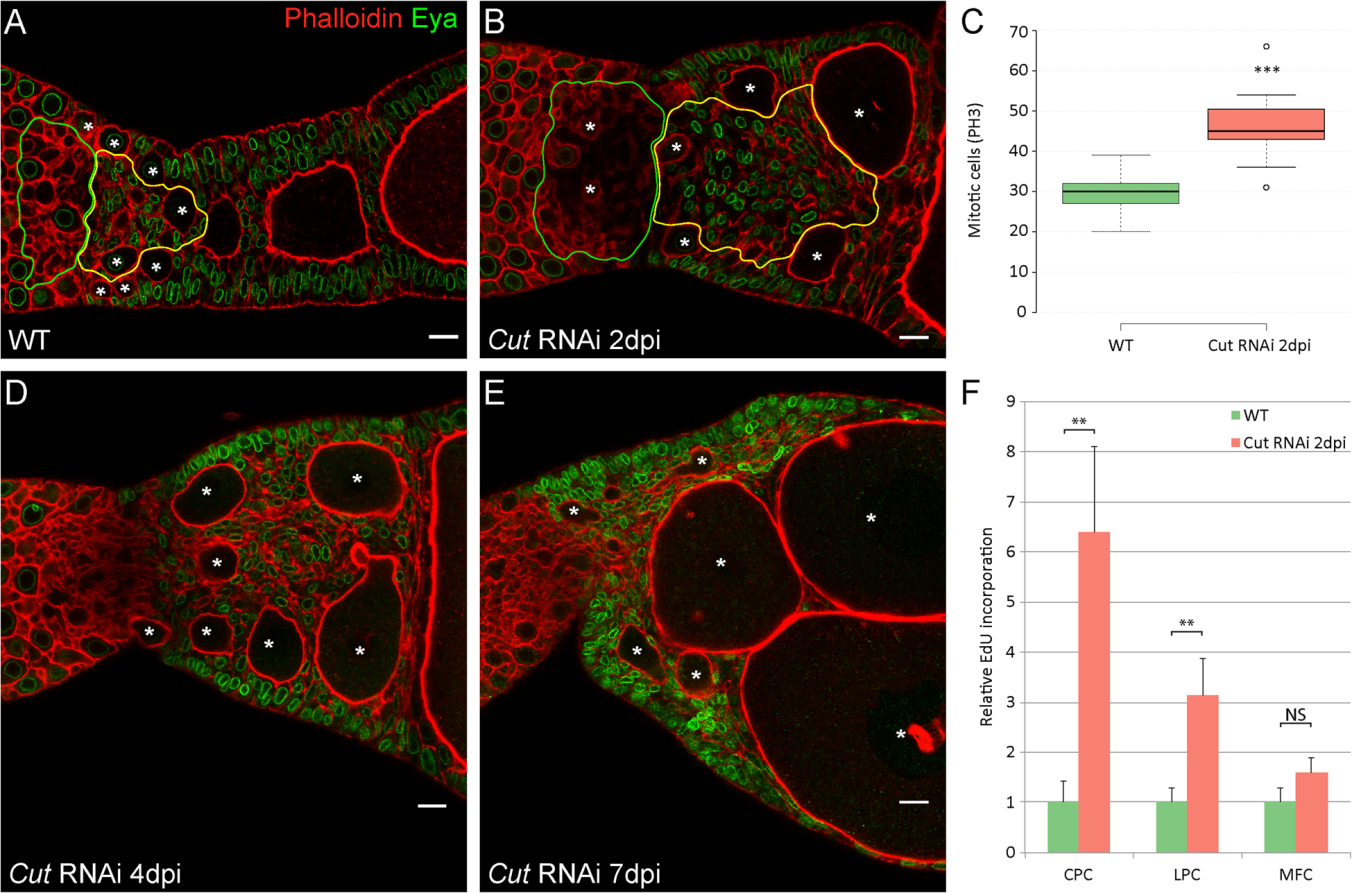
**a** Wild type ovariole stained with Phalloidin to visualize f-Actin (*red*) and an antibody against Eya (*green*). **b**, **d**, **e** RNAi phenotypes of *Cut* at indicated time points. The Knockdown of *Cut* results in the over proliferation of follicle cell populations with CPCs showing the strongest effect (**b**, *yellow outline*). Oocytes are not longer aligned, but positioned of side-by-side (*asterisks*). At subsequent dissections continuous growth of oocytes can be observed, resulting in the dislocation of CPCs (**d**–**e**). **c** Box plot of PH3 positive cells in WT (*green*) and *Cut* RNAi at 2dpi (*red*) shows increased mitosis rate after *Cut* knockdown. Centre lines show the medians; box limits indicate the 25th and 75th percentiles as determined by R software; whiskers extend 1.5 times the interquartile range from the 25th and 75th percentiles, outliers are represented by dots. *n* = 13, 11 sample points. **f** EdU signals in WT and *Cut* RNAi normalized to the mean of wild type. *Error bars* represent standard deviation. Central pre-follicular cells show the highest relative EdU incorporation (**f**). ** and *** *P* < 0.01 and *P* < 0.001 using a two-tailed t-test, respectively. Scale bar: 10 μm

First, we analysed the function of *Tc-cut* [41] during telotrophic oogenesis by adult RNAi. Ovaries were dissected two, four, and 7 days post injection and stained for Eya and pH 3 (Fig. 6). Already at 2dpi, follicle cell numbers have increased and as a consequence, early egg-chambers are miss-aligned and become separated by multiple layers of cells (Fig. 6b). Accordingly, high levels of Eya expression were observed across the entire follicle cell epithelium. Still, as judged by nuclear morphology, also *cut* RNAi follicle cells eventually enter endocycle (Fig. 6b). Hence, prolonged mitotic activity of follicle cells may contribute to the *cut* RNAi overproliferation phenotype.

As deduced by pH 3 staining (Fig. 6c, Additional file 1: Table S2), the number of mitotic follicle cell raised to more than 50 per ovariole. Interestingly, EdU incorporation revealed that central pre-follicular cells show the highest increase of mitotic activity, as compared to wild type (Fig. 6f). The expansion of this cell population may displace the oocytes and as a consequence, oocytes become arrange side-by-side, which eventually increases the overall diameter of the ovariole (Fig. 6b–e). Irrespective of the alignment phenotype, oocytes still grow and eventually, also enter vitellogenesis (Fig. 6d, e).

In *Drosophila*, Cut is required for cell differentiation and tissue homeostasis in embryonic and adult tissues [25–30]. On the one hand, Cut acts post-mitotic, i.e. regulates genes involved in cell differentiation and on the other hand, Cut was shown to maintain the normal mitotic cycle in follicle cells during *Drosophila* oogenesis. Also in mammals, Cut has been shown to be involved in regulating cell-cycle progression in some cell types [42–45]. While we did not observed any obvious impact of *Tribolium* cut on cell fate decision during follicle cell patterning in telotrophic *Tribolium* oogenesis, we found cut to act on cell-cycle regulation in *Tribolium* as well. However, *Tribolium* cut rather prevents (or balances) mitosis, as in absence of cut follicle cells continue to proliferate. Hence, even though cut has opposing effects on the mitotsis/endocycle switch in *Tribolium* and *Drosophila*, the general function of Cut in tissue homeostasis might well be conserved.

### *TC003132* acts upstream of Notch-signaling

**Fig. 7:**
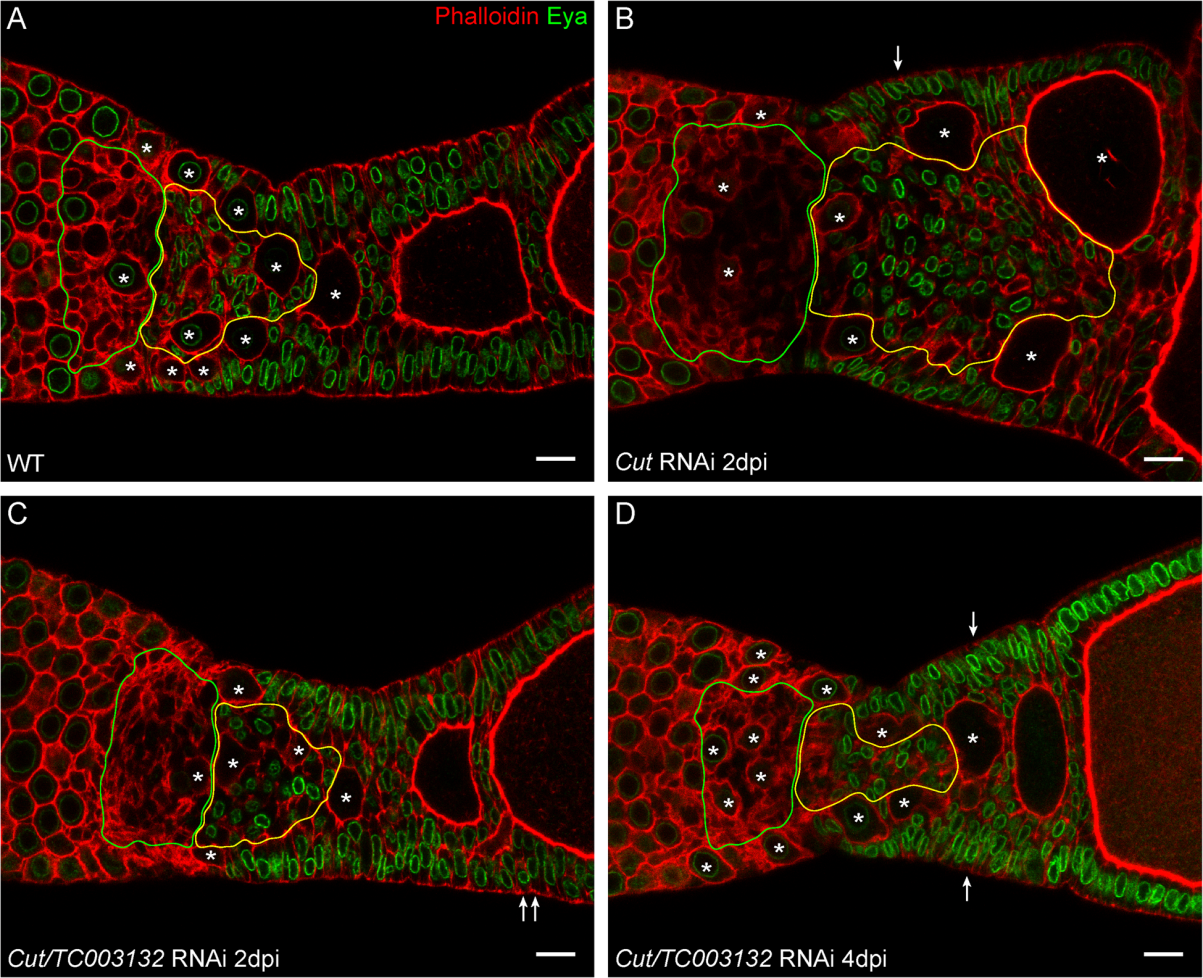
**a** Wildtype, **b** *Cut* RNAi, and **c**–**d** Double knockdown of *Cut* and *TC003132* at indicated time points. Two days after injection, only a mild *cut* phenotype is obvious. Minor over-proliferation can be observed (*arrows*, **c**). At 4dpi, multi-layered follicle cells (*arrows*) become more frequent, but other effects of *Cut* RNAi are supressed (**d**). Oocytes are marked by *asterisks*; Scale bar: 10 μm

**Fig. 8:**
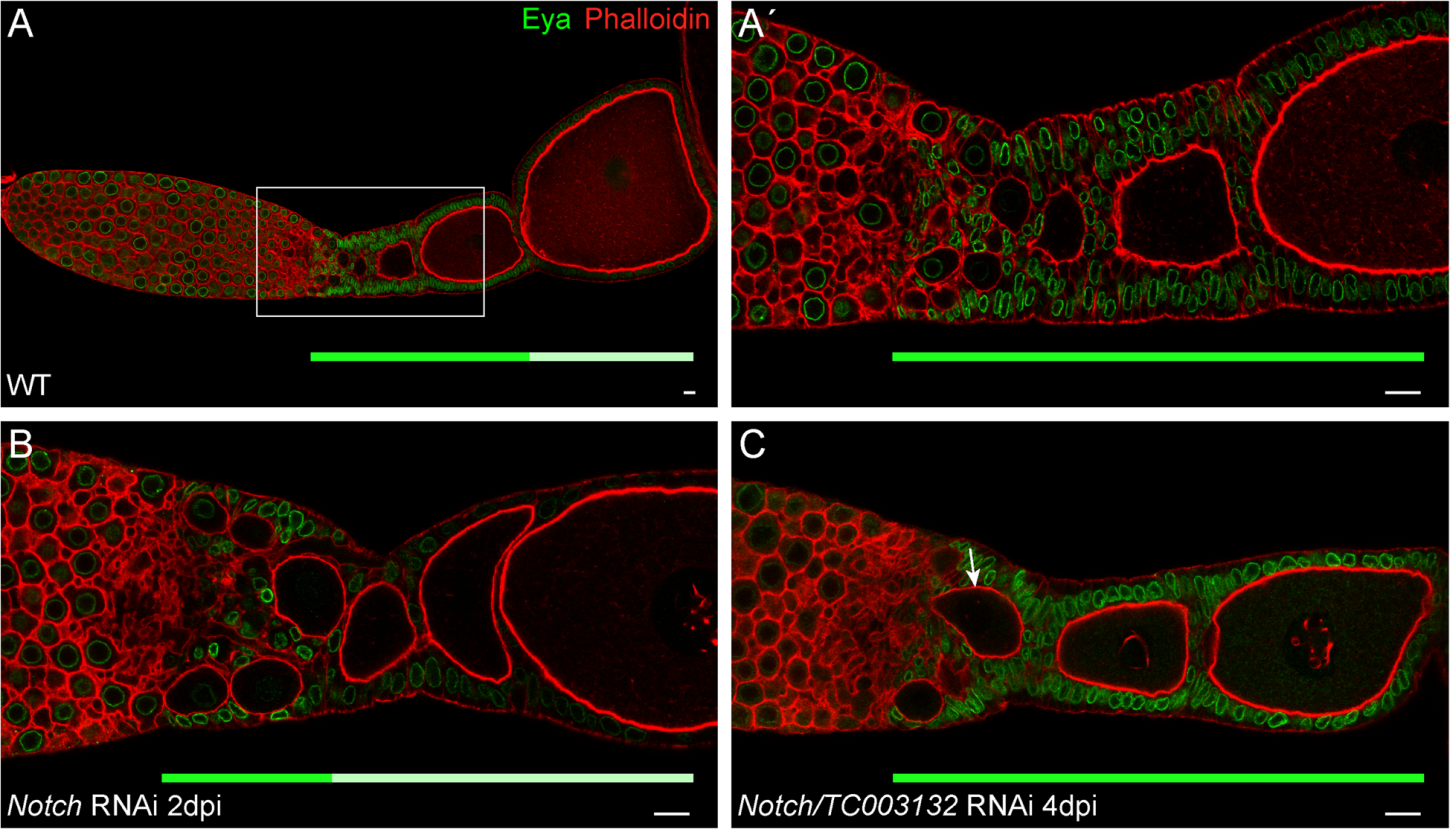
(**A**–**A′**) Wild type ovariole stained with Phalloidin to visualize f-Actin (*red*) and an antibody against Eya (*green*). Mitotic active follicle cells strongly express Eya, while cells that entered the endocycle cease Eya expression. (**A′**) Magnification of the indicated region in (**A**). (**B**) Knockdown of *Notch* results in the premature entry of the follicle cells into the endocycle. (**C**) Double RNAi of *Notch* and *TC003132* suppresses the effects of *Notch* RNAi in follicle cells: Eya expression is restored. Still, the germline shows signs of the Notch phenotype as prematurely growing oocytes are still obvious (**C**, *arrow*). *Solid green* and *light green bars* below ovarioles indicate regions of strong and weak Eya expression, respectively. Scale bar: 10 μm

In *Drosophila,* Cut expression is down-regulated by Notch signaling during the cell-cycle switch at stages 6–7 [30]. Notch-signalling in *Tribolium*, however, is required to maintain the mitotic cycle of somatic follicle cells, as upon *Tc-Notch* or *Tc-Delta* RNAi follicle cells cease mitosis and enter endocycle prematurely [15]. To further elucidate the functions of *TC003132* in regulating the mitotic activity of pre-follicular cells, we performed double RNAi experiments with *TC003132* and *cut* or *Notch* (Figs. 7 and 8).

First, we asked whether *Tc-Cut* and *TC003132* act in the same pathway or in parallel to each other. In the double knock down situation, the follicular epithelium is less disorganized and egg-chambers are aligned properly as compared to *cut* knockdown (Fig. 7). Also, the overall number of mitotically active cells in *TC003132* and *cut* double RNAi drops to almost wildtype levels (Additional file 1: Table S2). Thus, the depletion of *TC003132* counteracts the *Tc-cut* RNAi overproliferation phenotype.

Still, pre-follicular cell populations react somewhat different to the combined knock-down of *Tc-cut* and *TC003132*. In central pre-follicular cells, the *TC003132* phenotype is predominant (Fig. 7c). As deduced by Eya, CPCs are reduced in number, very similar to *TC003132* RNAi (Fig. 7c–d). In lateral pre-follicular cells, however, the *Tc-Cut* phenotype is still obvious. We observed an (albeit slight) increase of lateral follicle cells, resulting in a partially multi-layered follicular epithelium (Fig. 7, arrows in c–d). Hence, while *TC003132* counteracts the *Tc-cut* RNAi phenotype in most somatic cells, in central pre-follicular cell, *TC003132* is epistatic to *Tc-cut*.

In *Notch* RNAi, follicle cells cease the expression of Eya and switch to endocycle (Fig. 8, B) [15]. As a consequence, oocytes are not longer encapsulated properly and terminal follicle cells are absent. In addition, oocytes enter pre-vitellogenic growth prematurely. In the combined knock-down of *TC003132* and *Notch*, however, Eya is still expressed at high levels, indicating that simultaneous *TC003132* RNAi prevents follicle cells from entering the endocycle prematurely (Fig. 8, C). Thus, with respect to mitotic to endocycle transition, TC003132 acts upstream of Notch. However, since we observed growing oocytes in the transition zone (Fig. 8, arrow in C) TC003132 does not counteract all aspects of the *Notch* RNAi phenotypes.

## Conclusions

Our results indicate that *TC003132* is required for mitotic activity of pre-follicular cells. Previously, it was shown that *c1orf109*, which encodes a human CK2 substrate is involved in cancer cell proliferation by promoting G1 phase to S phase transition [46]. During oogenesis, G1 phase to S phase transition is required for both, mitotic and endocycle. Thus, the function of TC003132 in cell cycle control may very well be conserved. However, while central and lateral pre-follicular are (almost) equally affected upon *Notch* or *cut* depletion, only central pre-follicular cells are lost in *TC003132* RNAi. Lateral pre-follicular cells remain largely unaffected upon *TC003132* depletion and still express Eya.

These results indicate that *TC003132* might be required for the specification or maintenance of the central pre-follicular cell linage. Given that *TC003132* depleted pre-follicular cells neither react to mitosis or endocycle stimulating signals, TC003132 may be required to provide competence for differentiation cues. Interestingly, this may resemble the situation in *C. elegans* where CK2 is required to maintain the balance between proliferation and differentiation in the germ line [47]. While animals mutant for *kin10* – the worm homologue of the regulatory CK2β subunit – exhibit a smaller mitotic zone in the gonad, depletion in sensitized backgrounds also revealed an additional role for *kin10* in promoting the transition from mitosis to meiosis [47]. These dual functions might be carried out via different CK2 substrates.

The earliest effect of *TC003132* RNAi is characterized by the loss of Eya expression in a subpopulation of central pre-follicular cells. Strikingly, these cells resemble those slow dividing cells that retain the EdU signal. Given that these cells may resemble a follicle stem cell population, it is tempting to speculate that *TC003132* is required for the proliferation or maintenance of follicle stem cells in the telotrophic *Tribolium* ovary. In *Drosophila*, two actively dividing follicle stem cells (FSC) are present per ovariole and each cell produces approximately half of the follicle cells in the ovariole [48]. Escort cells are believed to provide niche factors that anchor the FSCs at the region 2a/2b border of the germarium and promote FSC self-renewal and differentiation [3]. Still, the number of stem cells may vary among different systems and organisms [49]. In the *Drosophila* testis, for instance, 7 to 15 germline stem cells are present and somatic hub cells serve as their niche. The number of stem cell in the Mouse hair follicle bulge is even higher, as more than 50 epithelial stem cells were identified [50]. Also multiple subtypes of niches exist. While in the *Drosophila* ovary only two cap cells serve as niche for the GSCs, mammalian subventricular zone (SVZ) neural stem cells are closely associated with multiple cell types, including astrocytes, neuroblasts, ependymal cells, endothelial cells and the basal-lamina (for a review see [51]). Our results suggest that also telotrophic ovary harbours a larger population of FSCs. Given that these putative FSCs are in direct contact to the somatic plug, we posit that this region of the telotrophic ovariole may serve as niche for the follicle stem cell population.

Still, more work will be required to identify the molecular mechanisms by which TC003132 regulates cell proliferation and/or differentiation of the follicle stem cell linage. Among others, the expression of *TC003132* remains to be elucidated. Unfortunately, ovariole-in-situ hybridisation is still not working robustly. To overcome these limitations, we generated several peptide antibodies against *TC003132*. However, for none of these antibodies we received conclusive results. In addition it remains to be elucidated, whether TC003132 is indeed a substrate of the casein kinase 2 (CK2). Given that CK2 is associated with cell survival, cell cycle progression, differentiation, and human cancer formation, the further analysis of *TC003132* may provide insights into a variety of important cellular processes, including the formation and maintenance of somatic stem cells.

## Additional file

Additional file 1:
**Table S1.** EdU positive cells in wildtype. **Table S2.** PH3 positive cells. **Table S3.** EdU positive cells in *TC003132* RNAi. **Table S4.** EdU positive cells in *Cut* RNAi. Methods. (DOCX 26 kb)
