# Supplementary material for: TC003132 is essential for the follicle stem cell lineage in telotrophic Tribolium oogenesis
Source: Front Zool. 2017 May 19;14:26. doi: 10.1186/s12983-017-0212-2 (PMC5438533; doi:10.1186/s12983-017-0212-2)
Supplement: Additional file 1: — Table S1. EdU positive cells in wildtype. Table S2. PH3 positive cells. Table S3. EdU positive cells in TC003132 RNAi. Table S4. EdU positive cells in Cut RNAi. Methods. (DOCX 26 kb) [file 12983_2017_212_MOESM1_ESM.docx]

Additional file 1

Table S1: EdU positive cells in wildtype

| **WT** | **Ovariole #** | **CPC** | **LPC** | **MFC** | **EFC** |
| --- | --- | --- | --- | --- | --- |
| **1 dpi** | 1 | 0 | 8 | 279 | 207 |
|  | 2 | 0 | 9 | 353 | 292 |
|  | 3 | 2 | 7 | 338 | 273 |
|  | 4 | 2 | 9 | 289 | 302 |
| **2 dpi** | 1 | 4 | 9 | 225 | 197 |
|  | 2 | 9 | 16 | 290 | 272 |
|  | 3 | 3 | 10 | 457 | 465 |
|  | 4 | 7 | 18 | 251 | 301 |
| **3 dpi** | 1 | 12 | 20 | 253 | 310 |
|  | 2 | 4 | 10 | 278 | 254 |
|  | 3 | 6 | 11 | 105 | 181 |
|  | 4 | 6 | 7 | 78 | 160 |
| **4dpi** | 1 | 8 | 4 | 32 | 18 |
|  | 2 | 5 | 2 | 8 | 5 |
|  | 3 | 4 | 6 | 10 | 2 |
|  | 4 | 10 | 7 | 23 | 12 |
| **7 dpi** | 1 | 2 | 0 | 0 | 0 |
|  | 2 | 4 | 2 | 1 | 0 |
|  | 3 | 6 | 2 | 1 | 0 |
|  | 4 | 7 | 2 | 0 | 0 |

Table S1: Amount of EdU positive cells in four different ovarioles at indicated time points after injection of 5mM EdU.

Table S2: PH3 positive cells

| **Ovariole #** | **WT** | **Cut RNAi 2dpi** | **TC003132 RNAi 5dpi** | **Cut/TC003132 RNAi 2dpi** |
| --- | --- | --- | --- | --- |
| **1** | 31 | 45 | 11 | 27 |
| **2** | 39 | 44 | 22 | 29 |
| **3** | 25 | 66 | 20 | 30 |
| **4** | 27 | 36 | 17 | 35 |
| **5** | 37 | 47 | 14 | 34 |
| **6** | 20 | 54 | 9 | 18 |
| **7** | 30 | 51 | 20 | 30 |
| **8** | 27 | 45 | 8 | 22 |
| **9** | 32 | 31 | 5 | 22 |
| **10** | 30 | 50 | 16 | 18 |
| **11** | 27 | 42 | 6 | 36 |
| **12** | 31 |  | 20 | 41 |
| **13** | 37 |  | 10 | 16 |
| **14** |  |  | 15 | 16 |
| **15** |  |  |  | 20 |
| **16** |  |  |  | 16 |

Table S2: Amount of PH3 positive cells dissected after indicated time points. *Cut* dsRNA 1µg/µl; *TC003132* dsRNA 2µg/µl

Table S3: EdU positive cells in *TC003132* RNAi

| **TC003132 RNAi** | **Ovariole #** | **CPC** | **LPC** | **MFC** | **EFC** |
| --- | --- | --- | --- | --- | --- |
| **3 dpi** | 1 | 6 | 15 | 298 | 235 |
|  | 2 | 7 | 12 | 262 | 281 |
|  | 3 | 12 | 19 | 347 | 343 |
|  | 4 | 18 | 16 | 308 | 250 |
| **5dpi** | 1 | 16 | 17 | 219 | 209 |
|  | 2 | 9 | 12 | 140 | 215 |
|  | 3 | 8 | 16 | 244 | 333 |
|  | 4 | 7 | 11 | 145 | 130 |
| **7 dpi** | 1 | 12 | 16 | 190 | 198 |
|  | 2 | 8 | 9 | 155 | 210 |
|  | 3 | 11 | 7 | 79 | 88 |
|  | 4 | 11 | 15 | 140 | 134 |

Table S3: Amount of EdU positive cells in four different ovarioles at indicated time points after simultaneous injection of 5mM EdU and 2µg/µl *TC003132* dsRNA.

Table S4: EdU positive cells in *Cut* RNAi

| **Cut RNAi** | **Ovariole #** | **CPC** | **LPC** | **MFC** | **EFC** |
| --- | --- | --- | --- | --- | --- |
| **2 dpi** | 1 | 25 | 34 | 616 | 0 |
|  | 2 | 30 | 39 | 389 | 430 |
|  | 3 | 50 | 58 | 533 | 637 |
|  | 4 | 42 | 35 | 395 | 493 |

Table S4: Amount of EdU positive cells in four different ovarioles at 2dpi after simultaneous injection of 5mM EdU and 1µg/µl *Cut* dsRNA.

**Methods**

*Ovary dissection and antibody staining*

Female beetles were anaesthetised on ice prior to dissection. Within 20 minutes as many ovaries were dissected and immediately fixed on ice in 5% formaldehyde diluted in PBS. After dissection the ovaries were fixed for an additional 30 minutes resulting in a total fixation time for each ovary between 30-50 minutes. After the fixation ovaries were rinsed once with PBS and twice with PBS containing 0.02% Tween-20 (PBT). The muscle sheath surrounding each ovariole was subsequently removed on a slide using insect needles glued to wooden skewers. It was taken care of that the ovaries always were covered with PBT. The cleaned ovarioles were collected on ice in PBT subsequently fixed for additional 15 minutes in 5% formaldehyde diluted in PBT at room temperature on a nutator. After the fixation was complete ovaries were rinsed twice in PBT followed by 3 washing steps in PBT/BSA (1x PBS, 1% BSA, 0.5% Triton X-100) for 15 minutes on a nutator at room temperature. The antibody was added to the ovaries in an appropriate concentration and incubated over night at 4°C. On the next day the ovaries were washed 3 times for 15 minutes with PBT/BSA before the fluorescently labelled secondary antibody was added in an appropriate concentration and again incubated over night at 4°C. After the incubation ovaries were washed again 3 times for 15 minutes at room temperature with PBT/BSA and subsequently stored at 4°C for up to 4 days. For imaging the ovaries were separated and mounted on a slide in a 1:1 mixture of glycerol and 1x PBS, covered by a coverslip with putty stands and analysed at the microscope.
